# Soluble expression of recombinant midgut zymogen (native propeptide) proteases from the *Aedes aegypti* Mosquito Utilizing *E. coli* as a host

**DOI:** 10.1186/s12858-018-0101-0

**Published:** 2018-12-18

**Authors:** James T. Nguyen, Jonathan Fong, Daniel Fong, Timothy Fong, Rachael M. Lucero, Jamie M. Gallimore, Olive E. Burata, Kamille Parungao, Alberto A. Rascón

**Affiliations:** Department of Chemistry, Duncan Hall 612, One Washington Square, San José State University, San José, CA 95192 USA

**Keywords:** *Aedes aegypti*, Midgut, Proteases, Zymogen, Soluble expression, Recombinant protein, *Escherichia coli*, Disulfide bond/bridge

## Abstract

**Background:**

Studying proteins and enzymes involved in important biological processes in the *Aedes aegypti* mosquito is limited by the quantity that can be directly isolated from the mosquito. Adding to this difficulty, digestive enzymes (midgut proteases) involved in metabolizing blood meal proteins require a more oxidizing environment to allow proper folding of disulfide bonds. Therefore, recombinant techniques to express foreign proteins in *Escherichia coli* prove to be effective in producing milligram quantities of the expressed product. However, with the most commonly used strains having a reducing cytoplasm, soluble expression of recombinant proteases is hampered. Fortunately, new *E. coli* strains with a more oxidizing cytoplasm are now available to ensure proper folding of disulfide bonds.

**Results:**

Utilizing an *E. coli* strain with a more oxidizing cytoplasm (SHuffle® T7, New England Biolabs) and changes in bacterial growth temperature has resulted in the soluble expression of the four most abundantly expressed *Ae. aegypti* midgut proteases (AaET, AaSPVI, AaSPVII, and AaLT). A previous attempt of solubly expressing the full-length zymogen forms of these proteases with the leader (signal) sequence and a modified pseudo propeptide with a heterologous enterokinase cleavage site led to insoluble recombinant protein expression. In combination with the more oxidizing cytoplasm, and changes in growth temperature, helped improve the solubility of the zymogen (no leader) native propeptide proteases in *E. coli*. Furthermore, the approach led to autocatalytic activation of the proteases during bacterial expression and observable BApNA activity. Different time-points after bacterial growth induction were tested to determine the time at which the inactive (zymogen) species is observed to transition to the active form. This helped with the purification and isolation of only the inactive zymogen forms using Nickel affinity.

**Conclusions:**

The difficulty in solubly expressing recombinant proteases in *E. coli* is caused by the native reducing cytoplasm. However, with bacterial strains with a more oxidizing cytoplasm, recombinant soluble expression can be achieved, but only in concert with changes in bacterial growth temperature. The method described herein should provide a facile starting point to recombinantly expressing *Ae. aegypti* mosquito proteases or proteins dependent on disulfide bonds utilizing *E. coli* as a host.

**Electronic supplementary material:**

The online version of this article (10.1186/s12858-018-0101-0) contains supplementary material, which is available to authorized users.

## Background

The *Aedes aegypti* mosquito is responsible for the transmission of the Zika, Dengue, and Yellow Fever flaviviruses, as well as the Chikungunya alphavirus. The female mosquito may become infected with each virus upon imbibing a blood meal from an infected human host. The uptake of a blood meal is required to produce the necessary nutrients needed for egg production, without it the mosquito life cycle would cease [1]. Unfortunately, it is this blood meal acquisition step that allows the transmission of viruses from an infected female mosquito to an uninfected human host. Each viral infection leads to many common flu-like symptoms like fever, headache, rash, joint and muscle pain, but in severe cases, hemorrhagic stages and even death can occur (as is the case with Dengue and Yellow Fever) [2]. For Chikungunya, infection may lead to long-lasting joint pain that may last several weeks or years [3]. Currently, however, the biggest threat to people around the world is Zika. Zika viral infection has been directly linked to microcephaly in newborn babies and linked to Guillian-Barré syndrome [4], a condition in which the body’s own immune systems attacks the nerves.

Brazil was the first country in the Americas to report the presence of Zika in 2015, but transmission has spread beyond South America, to Mexico and the United States, with local transmission observed in both Florida and Texas in the last few years [4, 5]. This rapid expansion is believed to be due to the feeding behavior of the *Ae. aegypti* mosquito, preferring to feed on humans and preference to live near people in urban areas, but also to warmer climates creating ideal environmental conditions for the mosquito to thrive [6]. Although vaccines are available to combat the Yellow Fever virus (YFV-17D) and the Dengue virus (Dengvaxia, CYD-TDV), there are no licensed vaccines available to combat the Zika and Chikungunya viruses [7, 8]. Of the two vaccines, vaccination with YFV-17D and its sub-strains lead to lifelong immunity, as for Dengvaxia, recent studies have shown that Dengvaxia vaccination has led to severe illness and hospitalizations on people who have never been exposed to Dengue [9, 10]. Regardless, however, the World Health Organization still recommends the use of the vaccine in highly endemic regions [11]. An alternative approach, which is still the leading approach to combat mosquito-borne viral infections and transmission, is through vector control strategies, with the most common being the use of insecticides or larvicides [6, 12]. However, the use of organophosphates, pyrethroids, carbamates, and organochlorides to combat the adult and larval *Ae. aegypti* are leading to resistance, eventually becoming more ineffective [6]. This had led to the integration of simultaneous intervention methods using synthetic insecticides and repellents, insecticidal nets, sterile insect techniques and transgenic mosquitoes, which on their own, are not as effective as the simultaneous use of them [12]. Therefore, there is a need to explore new and more efficient vector control strategies.

One potential vector control strategy is to focus on the digestive enzymes (proteases) involved in the breakdown of blood meal proteins [1, 13–16]. Upon ingestion of a blood meal, several midgut endo- and exo-proteases are released at different times during the biphasic blood meal digestion period [17], which aids in producing amino acids and oligopeptides needed for egg production and other metabolic processes [1]. It is important to note, that these proteases are only expressed after a blood meal has been imbibed, with some proteases being regulated at the protein translational level [14, 18] and others at the transcriptional level [19]. In 2009, Isoe et al. utilized RNAi knockdown studies to functionally identify candidate midgut proteases responsible for the breakdown of blood meal proteins in the *Ae. aegypti* mosquito. In their findings, knockdown of three abundant midgut proteases (AaSPVI, formerly known as 5G1, NCBI Accession # GQ398048), AaLT (NCBI Accession # M77814), AaSPVII (NCBI Accession # GQ398049)) led to a decrease in fecundity compared to FLUC controls [1]. The *Ae. aegypti* mosquito is highly dependent on these proteolytic enzymes to achieve maximal fecundity, so targeting the proteases responsible for degrading the major blood meal proteins should inhibit the production of the necessary nutrients for the egg-laying process, reducing the mosquito population and leading to a reduction in viral pathogen transmission. What makes this process an ideal target is the fact that ~ 80% of the blood meal is composed of mostly protein and only a handful of midgut proteases involved to break these down into necessary amino acid and poly-peptide nutrients [1].

Mosquito control is key in slowing the spread of mosquito-borne diseases, especially in areas with high pathogen transmission. However, before validating midgut proteases as a potential vector control strategy, we must first fully understand how individual midgut proteases digest blood meal proteins. This involves in vitro biochemical studies using recombinant technology because one can achieve high levels of an enzyme product that would otherwise be difficult to isolate, especially if the source is only available in small quantities [20]. Initial attempts at recombinantly expressing mosquito midgut proteases using *Escherichia coli* led to insoluble expression (inclusion bodies), which were isolated and purified using a denaturation/renaturation strategy [15]. This approach did lead to active recombinant midgut proteases, but rather than produce recombinant proteases with the native (wild type) propeptide region, a heterologous enterokinase cleavage site was engineered to allow for the activation of the proteases in vitro. With engineering of the SHuffle *E. coli* strain (New England Biolabs), with a more oxidizing cytoplasm, the four most abundant proteases were successfully solubly expressed in a bacterial system. This strain of *E. coli* has deletions in thioredoxin reductase (*trxb*) and glutathione reductase (*gor*), proteins that promote a reducing cytoplasm, similarly to Origami cells manufactured by Novagen [21]. However, rather than overexpressing the disulfide bond isomerase A (DsbA) chaperone (Origami cells), SHuffle cells overexpress the DsbC periplasmic chaperone in the cytoplasm, which specifically isomerizes mis-oxidized proteins to their native states, but also results in more soluble expression compared to periplasmic expression systems (fully reviewed and explained in [21]). Therefore, here we describe the cloning and successful recombinant soluble expression of wild type zymogen (inactive no leader sequence) forms of the most abundant *Ae. aegypti* mosquito midgut proteases using SHuffle *E. coli* cells. The method described should provide a facile starting point to determine the conditions needed to recombinantly solubly expressing mosquito proteases or other eukaryotic proteins dependent on disulfide bonds using bacteria as a host.

## Methods

The approach to solubly expressing recombinant proteolytic enzymes in *E. coli* are described for the most abundant midgut proteases (AaET, NCBI Accession # X64362), AaSPVI, AaSPVII and AaLT), each dependent on three disulfide bonds for structure and function. However, the successful approach described may be applicable to other proteases or proteins dependent on disulfide bond formation.

### Chemicals

Reagent grade (or better) Tris Base (2-amino-2-(hydroxymethyl)-1,3-propanediol), calcium chloride, hydrochloric acid, dithiothreitol (DTT), imidazole, isopropyl-β-D-thiogalactopyranoside (IPTG), Nα-benzoyl-DL-arginine-4-nitroanilide (BApNA), kanamycin, Luria Broth, and Terrific Broth were purchased from Fisher Scientific (Thermo Fisher Scientific, Waltham, MA). To visualize protease expression of bacterial lysates and of concentrated purified proteases, 4–12% (gradient) or 12% NuPAGE™ Bis-Tris Protein Gels (Invitrogen, Carlsbad, CA) were used and stained with either InVision™ His-Tag In-Gel Stain (Invitrogen #LC6030), SimplyBlue™ Safe Stain (Invitrogen #LC6065) or both. For western blots, AaET-specific and other custom antibodies were purchased from GenScript (Piscataway, NJ) and have been previously described [1, 15].

### Cloning and engineering of recombinant DNA plasmid constructs

Full-length zymogen plasmid constructs with the signal (leader) sequence were originally cloned into the pET vector system (Novagen, Madison, WI), as described in [15]. However, for the purpose of this study, the leader sequence in each of the proteases was removed. SignalP 4.1 [22] was used to identify the leader sequence and primers were designed for cloning of the no leader zymogen mosquito midgut proteases (Table 1) (from here on, proteases without the leader (no leader proteases) will be referred as NL, e.g.*,* AaET-NL). Primers with the NdeI and HindIII restriction cleavage sites were included for cloning of AaET-NL, AaSPVI-NL, and AaSPVII-NL into the pET28a vector (Novagen #69864–3), while NdeI and XhoI sites were included for AaLT-NL. The mosquito protease genes of interest were PCR amplified using the GoTaq® Green Master Mix (Promega #M7122, Madison, WI), following the manufacturer’s protocol. Once engineered, plasmid constructs were verified by DNA sequencing (ELIM Biopharmaceuticals, Inc., Hayward, CA).

**Table 1:** Primers designed and used for PCR amplification and cloning of zymogen (no leader) *Ae. aegypti* mosquito midgut proteases. Primers were purchased from ELIM Biopharmaceuticals, Inc. The melting temperature (T_M_) of the primer sequence that anneals to the gene of interest was estimated using NetPrimer (Premier Biosoft, Palo Alto, CA). The restriction enzyme used for each primer are underlined and in bold

Protease	Primer	Primer Sequence	T_M_ (°C)
AaET	AaET-Zym-pET-Fwd	5’-AAAAA**CATATG**GCAACGCTGTCCAGCGGTC-3’	64.55
AaET	AaET-Zym-pET-Rev	5’-AAAAA**AAGCTT**ATTAAACCTCGGAAACCTCTCGGA-3’	64.24
AaSPVI	AaSPVI-No Leader-Fwd	5′ –AAAAA**CATATG**GCTTCAACCGGTGGTTTGC– 3’	61.1
AaSPVI	AaSPVI-Zym-pET-Rev	5′ –AAAAA**AAGCTT**ATTACAATCCACTGACCTCCTGG– 3’	59.09
AaSPVII	AaSPVII-Zym-pET-Fwd	5’-AAAAA**CATATG**CTATCAACCGGATTCCATCCGC-3’	65.36
AaSPVII	AaSPVII-Zym-pET-Rev	5’-AAAAA**AAGCTT**ATTAAACTCCACTGACTTCCGCCA-3’	63.72
AaLT	AaLT-Zym-pET-Fwd	5’-AAAAA**CATATG**TTCCCATCGTTGGACAACG-3’	59.59
AaLT	AaLT-Zym-pET-Rev	5’-AAAAA**CTCGAG**TTATTACAGTCCAGTCTTCTGCTTGA-3’	57.11

### Expression of recombinant proteases using SHuffle® (NEB) *E. coli* cells and Bis-Tris gel analysis

The most commonly used bacterial strain for expression of genes cloned into the pET vector system are the BL21(DE3) and *E. coli* K12 lineage strains [20]. These strains are the go to strains for initial screening and to ensure that the bacterial host can express the eukaryotic gene of interest. The problem however, is that these strains have a reducing cytoplasm caused by the activities of thioredoxin reductase and glutathione reductase [23]. Because of this, the initial attempt at solubly expressing the four abundant midgut proteases failed leading to insoluble expression (inclusion bodies) [15]. The disulfide bridges in these proteases were unable to form (all proteases in this study depend on three disulfide bonds for structure and function), therefore destabilizing the enzymes and promoting aggregation. To overcome this, we turned to a BL21(DE3) derivative known as SHuffle® T7 Express Competent *E. coli* (New England Biolabs #C3029J, Ipswich, MA). Plasmid constructs (25 to 50 ng pDNA) were transformed following the manufacturer’s protocol. For small or large-scale growth expression, a single colony from transformed overnight plates was selected to set overnight liquid cultures in LB media supplemented with kanamycin (30 μg/ml) and set in a 30 °C shaker (250 rpm) for 16 to 18 h. The optical density at 600 nm (OD_600nm_) of the overnight cultures was determined using a spectrophotometer and used as the starter culture to initiate growth at a starting OD_600nm_ ~ 0.05 in fresh, autoclaved media. We have found success utilizing Terrific Broth (TB) as the main growth media for all growth experiments, but any rich media containing extra carbon sources should suffice. All growths were then set in a 30 °C shaker (250 rpm) and the OD_600nm_ monitored until reaching an OD_600nm_ ~ 0.5–1.0 (middle of the log phase of bacterial growth). Once the density of the bacterial cells reached the proper OD_600nm_, the cells were induced with 0.1 mM IPTG (this concentration of IPTG was utilized based on the findings in [21]). However, different post-induction growth temperatures were tested in order to produce soluble recombinant proteases. Initial growth experiments utilized the manufacturer’s recommended 30 °C post-induction temperature, but in subsequent growth experiments, lower temperatures (23 °C, 15 °C, and 10 °C) were tested [21]. All growth experiments were grown to reach an OD_600nm_ ~ 0.5–1.0 at 30 °C, but before induction and growth at the lower temperatures, the growths were cooled on ice for 5 min, then induced and set at the respective lower post-induction temperature. Specifically, for AaET-NL and AaSPVII-NL, soluble expression was observed when cells grown at 23 °C and 15 °C (250 rpm) after induction with IPTG. As for AaSPVI-NL and AaLT-NL, soluble expression resulted when cells grown at 15 °C and 10 °C (250 rpm). Each growth experiment was repeated independently a minimum of three times. A no induction control was set as described above for AaET, AaSPVI, and AaSPVII, grown at 30 °C, set on ice when cells reached an OD_600nm_ ~ 0.5–1.0, and grown at 15 °C.

SDS-PAGE analysis was utilized to detect protease expression of 1 ml samples collected at pre- and post-induction (time points varied depending on the temperature). The 1 ml samples collected were centrifuged (full speed for 3 min at 4 °C on a tabletop centrifuge), supernatant discarded, and pellets stored at − 20 °C until use. Each pellet was solubilized in 20 mM Tris-HCl, pH 7.2, and sonicated at 25% amplification for 10 s each (total of three cycles) on ice utilizing a micro-tip cell disruptor (Fisher Model 505 Sonic Dismembrator). A total protein sample (0.02 ml) was collected before centrifugation, then centrifuged at 13,000 rpm (4 °C) for 5 min. Once centrifuged, a 0.02 ml soluble (supernatant) sample was collected. All samples were treated with 6x SDS sample buffer and denatured at 95 °C for 4 min. Samples were loaded on to 4–12% or 12% Bis-Tris gels in the presence of prestained PageRuler™ protein ladder (Thermo Scientific #26616 or #26619), followed by staining with InVision™ His-Tag In-Gel Stain and/or SimplyBlue™ Safe Stain. For low-level detection and identification of AaET, an AaET-specific antibody was used and the western blot protocol described in Rascon et al. [15] was followed.

### In vitro BApNA activity assays of bacterial crude lysates

For detection of trypsin-like activity, BApNA was used as the synthetic chromogenic substrate, as described in [15]. However, the final reaction conditions used were 20 mM TRIS-HCl pH 7.2 + 10 mM CaCl_2_, 1 mM BApNA, and 20 μl of soluble pre- and post-induction crude lysates collected from the growth experiments (1 ml total reaction volume). As a control, non-induced bacterial crude lysates were set as described. Each assay was repeated independently a minimum of three times and plotted using GraphPad Prism software (mean values ± SEM).

### Purification of Solubly expressed recombinant midgut proteases

With the success of solubly expressing recombinant midgut proteases, we proceeded to FPLC purify AaSPVII-NL and AaLT-NL using a 5 ml HisTRAP FF Nickel column (GE HealthCare #17–5255-01, Chicago, IL) on an AKTA Pure L1 Chromatography System (GE HealthCare). For purification, cell paste was solubilized in ice-cold 20 mM TRIS-HCl, pH 7.2 + 250 mM NaCl + 10 mM Imidazole + 2 mM DTT (Buffer A) (1 g frozen cell paste per 5 ml buffer ratio). The lysate was then sonicated at 35% amplification for 15 min on ice (15 s on/30 s off cycles), followed by centrifugation at 16,000 rpm (4 °C) for 35 min. The clear crude lysate was loaded on to an equilibrated HisTRAP Nickel column and washed with 10 column volumes (CV) of Buffer A, followed by a three-step linear elution gradient (10%B for 3 CV, 30%B for 6 CV, 50%B for 5 CV) with 20 mM TRIS-HCl, pH 7.2 + 250 mM NaCl + 500 mM Imidazole + 2 mM DTT (Buffer B). Fractions (1.5 ml each) containing the protease of interest were collected (detected by protein gel analysis) and pooled together. The pooled purified fractions were then dialyzed in 2 L 50 mM Sodium Acetate pH 5.2 + 2 mM DTT (twice) at 4 °C to remove excess imidazole and NaCl. The next day, the dialyzed protease was concentrated using an Amicon Ultra-15 Centrifugal Filter (10 kDa NMWL) (Millipore Sigma #UFC901024, St. Louis, MO), following the manufacturer’s protocol. The final concentrated protease was aliquoted, flash frozen in liquid nitrogen, and stored at − 80 °C. The concentration of the protease was estimated using the Pierce™ BCA Protein Assay Kit (Thermo Fisher #23227). As a final step, different quantities of protease were loaded on to a Bis-Tris protein gel in order to visualize excess contaminants. We are currently in the process of purifying AaET-NL and AaSPVI-NL.

## Results

In attempting to recombinantly express any protein or enzyme, *E. coli* is the first and most convenient host organism to use. There are several review articles that focus on overcoming difficulties in recombinant protein expression, as well as optimizing the conditions to improving the solubility of recombinantly expressed protein (see [20, 24, 25]). Of the many suggestions offered, the five that can easily be modified are the type of bacterial cells, type of specific vectors with fusion tags, type of rich media to use, IPTG concentration, and the temperature at which cells are grown. In attempting to recombinantly express eukaryotic proteases in bacteria, all of these factors come in to play to ensure the expression of active, soluble proteases. However, we have found that expression of the zymogen form of *Ae. aegypti* midgut proteases prove to be no problem when using the BL21(DE3) or Rosetta2(DE3) cell strains [15]. The bacterial cells are not affected by the expression of the proteases and leads to high concentrations, but as insoluble inclusion bodies. The proteases require three disulfide bonds for structure and function, and with a reducing cytoplasm, proper disulfide bond formation is difficult. Therefore, we focused on SHuffle® T7 *E. coli* cells with a more oxidizing cytoplasm and bacterial growth temperature to solubly express the *Ae. aegypti* midgut proteases.

### Soluble expression of zymogen (native propeptide) midgut proteases without the leader sequence

**Fig. 1:**
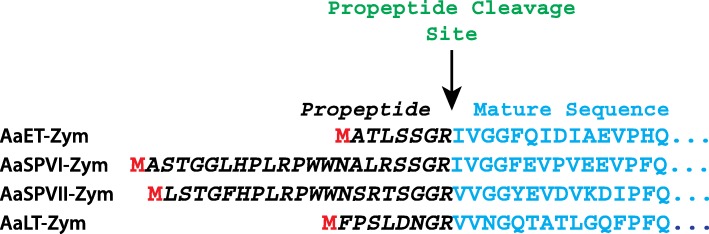
Amino acid sequences of *Ae. aegypti* midgut zymogen (no leader) proteases. In order to improve solubility of recombinant proteases, the leader (signal) sequence was removed to produce the no leader zymogen as shown. Since the genes were cloned into the pET28a vector (utilizing the NdeI restriction site, CATATG), the resulting recombinant proteases will contain an N-terminal Methionine (shown in red), as well as the his_6_-tag linker (MGSSHHHHHHSSGLVPRGSH) upstream of the Met group (shown in red). The arrow points to the propeptide cleavage site required to activate the zymogen to the active mature protease

At the time of initial cloning and expression of full-length *Ae. aegypti* midgut zymogen proteases with the leader (signal) sequence [15], specially designed bacterial cells with a more oxidizing cytoplasm were not as commercially available and limited. Therefore, the attempts of expressing soluble proteases were hampered due to lack of properly oxidized disulfide bond formation. A few years ago, new bacterial cells (SHuffle® T7, NEB) with a more oxidizing cytoplasm, along with the expression of a disulfide bond isomerase (DsbC), a protein that aids in correcting the folding of mis-oxidized disulfide bonds, became available [21]. These SHuffle® cells are a bit superior compared to Origami (Novagen) because DsbC does not oxidize just any available cysteine residue, only those mis-oxidized proteins that have the core hydrophobic residues exposed are targeted [21]. Furthermore, amino acid sequence analysis and literature search revealed that the leader (signal) sequence in eukaryotic proteins, which is fairly hydrophobic, could lead to aggregation upon expression in bacteria [26]. To overcome these issues, we engineered the no leader (signal) sequence zymogen plasmid constructs of AaET, AaSPVI, AaSPVII, and AaLT (Fig. 1), then transformed into SHuffle® T7 cells and grown in TB media at various temperatures.

**Fig. 2:**
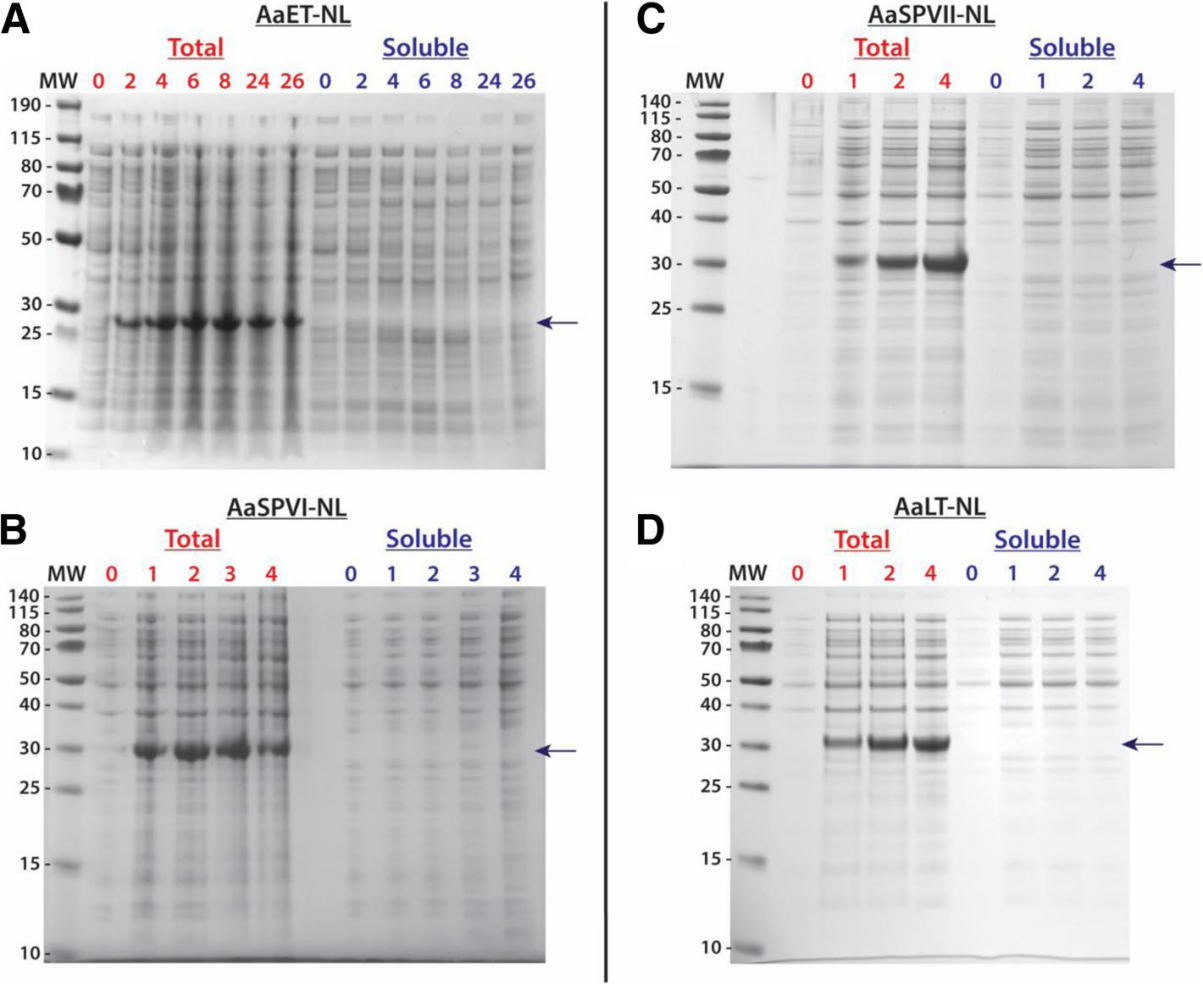
Initial attempt at solubly expressing recombinant midgut proteases in SHuffle® T7 Express Competent *E. coli* cells (NEB). For each growth experiment, TB media and a 30 °C growth temperature was used. Cells were induced with 0.1 mM IPTG when reaching the log phase (OD_600nm_ ~ 0.5–1.0). Samples were collected at the given time points (in hours) and prepared for SDS-PAGE analysis. The MW ladder is in kilo-Daltons (kDa). In all cases, the arrow indicates where the expected soluble over-expressed protease should appear. However, all proteases under these conditions were expressed insolubly, only observed in the total samples. **a** 4–12% BIS-TRIS gel over-expression of AaET grown for a total of 26 h. The MW of the his_6_-tagged AaET-NL zymogen is ~ 27.0 kDa. **b** 12% BIS-TRIS gel over-expression of AaSPVI grown for a total of 4 h. The MW of the his_6_-tagged AaSPVI-NL zymogen is ~ 28.7 kDa. **c** 12% BIS-TRIS gel over-expression of AaSPVII grown for a total of 4 h. The MW of the his_6_-tagged AaSPVII-NL zymogen is ~ 28.7 kDa. **d** 12% BIS-TRIS gel over-expression of AaLT grown for a total of 4 h. The MW of the his_6_-tagged AaLT-NL zymogen is ~ 27.6 kDa

**Fig. 3:**
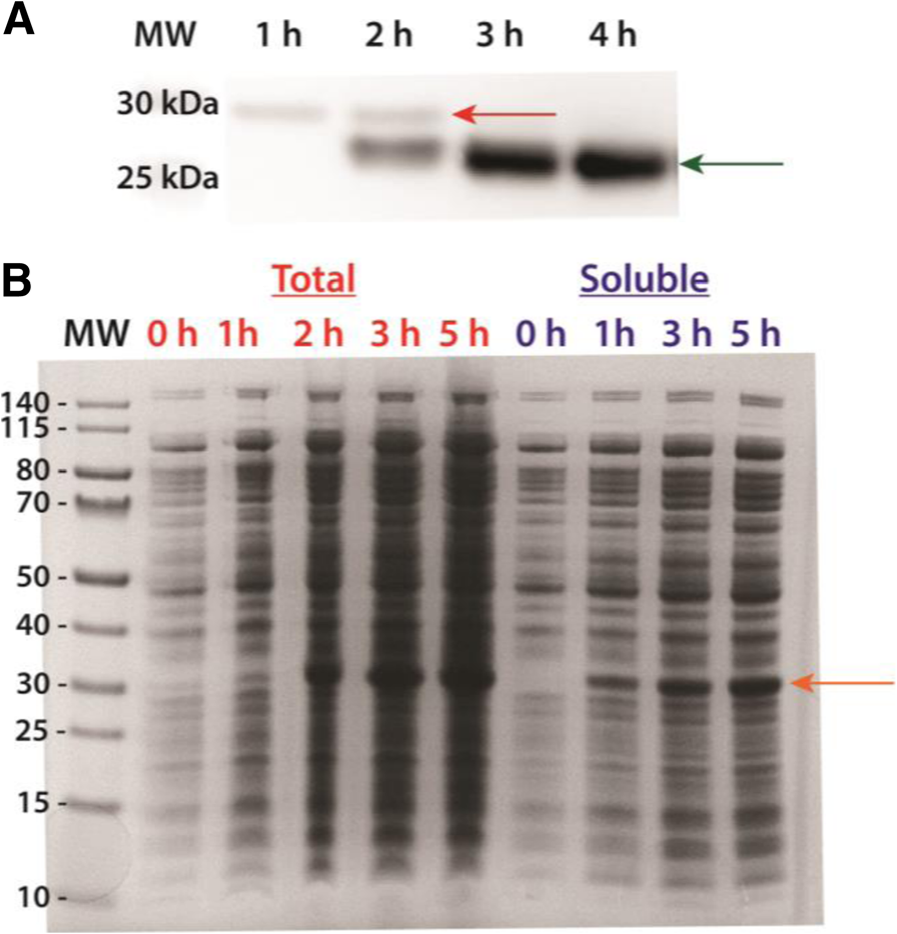
Soluble expression of recombinant AaET-NL and AaSPVII-NL zymogen proteases grown in TB media at 23 °C post-induction (induced with 0.1 mM IPTG). Plasmid constructs were transformed into SHuffle® T7 Express Competent *E. coli* cells (NEB). The MW ladder is in kilo-Daltons (kDa). **a** Western blot analysis utilizing an AaET-specific antibody of soluble samples collected from the growth and expression of AaET (a total of 4 h post-induction). The zymogen (inactive form of the protease) is observed in the first 2 h (MW ~ 27.0 kDa, red arrow), but a second species hypothesized to be the active mature form begins to appear at the two-hour time-point (MW ~ 22.4 kDa, green arrow) while the zymogen completely disappears by the third hour post-induction. **b** Large scale expression analysis of AaSPVII-zymogen grown for a total of 5 h post-induction. A single band at ~ 28.7 kDa (orange arrow) is observed to be increasing over time after induction with no observable band present in both the total and soluble pre-induction samples (*t* = 0 h)

To express the gene of interest, the manufacturer suggests a starting temperature of 30 °C, which is lower than the ideal *E. coli* growth conditions of 37 °C. This is due to the sensitivity of SHuffle® T7 cells to temperature, which is caused by deletions in *trxb* and *gor* [21]. Therefore, NL midgut protease expression started at 30 °C and induction with 0.1 mM IPTG. Initial soluble expression attempts at this temperature and IPTG concentration led to insoluble expression, as seen in Fig. 2. However, since the goal is to produce soluble proteases, in reading the literature [20, 24, 25], and recommendations from the manufacturer [21], the next condition altered to improve solubility was temperature. It is important to note, that the conditions to solubly expressing *Ae. aegypti* midgut proteases were investigated separately and determined to vary among the four different proteases. Caution should be taken when investigating the best conditions to solubly express eukaryotic proteases and change a single variable at a time. Hence, for these studies temperature was the next obvious step, and we have determined the optimal bacterial growth temperature conditions that led to the best soluble expression of each mosquito protease. For all growths, Terrific Broth and an initial growth temperature (pre-induction) at 30 °C were used to reach an OD_600nm_ ~ 0.5–1.0. This was done to ensure a lag to mid-log phase of 3.5 h. If grown at a lower (colder) temperature before induction, the log phase would take longer to achieve. Once the proper optical density observed, the bacterial growth temperatures were reduced (post-induction). The first temperature attempted was 23 °C, and as shown in Fig. 3a, the soluble expression of AaET-NL zymogen was observed between one to 2 h post-induction. Interestingly, under the conditions tested, the enzyme seems to auto-catalyze converting the inactive zymogen to the active mature form of the enzyme. In the western blot shown, an AaET-specific antibody was utilized to detect the expression of the protease of interest leading to the observation of two bands. BApNA activity was tested, but no activity observed. Another protease that expressed solubly at 23 °C was AaSPVII (Fig. 3b). Unlike AaET, AaSPVII is optimally solubly expressed within 5 h post-induction with 0.1 mM IPTG and only a single band is observed.

**Fig. 4:**
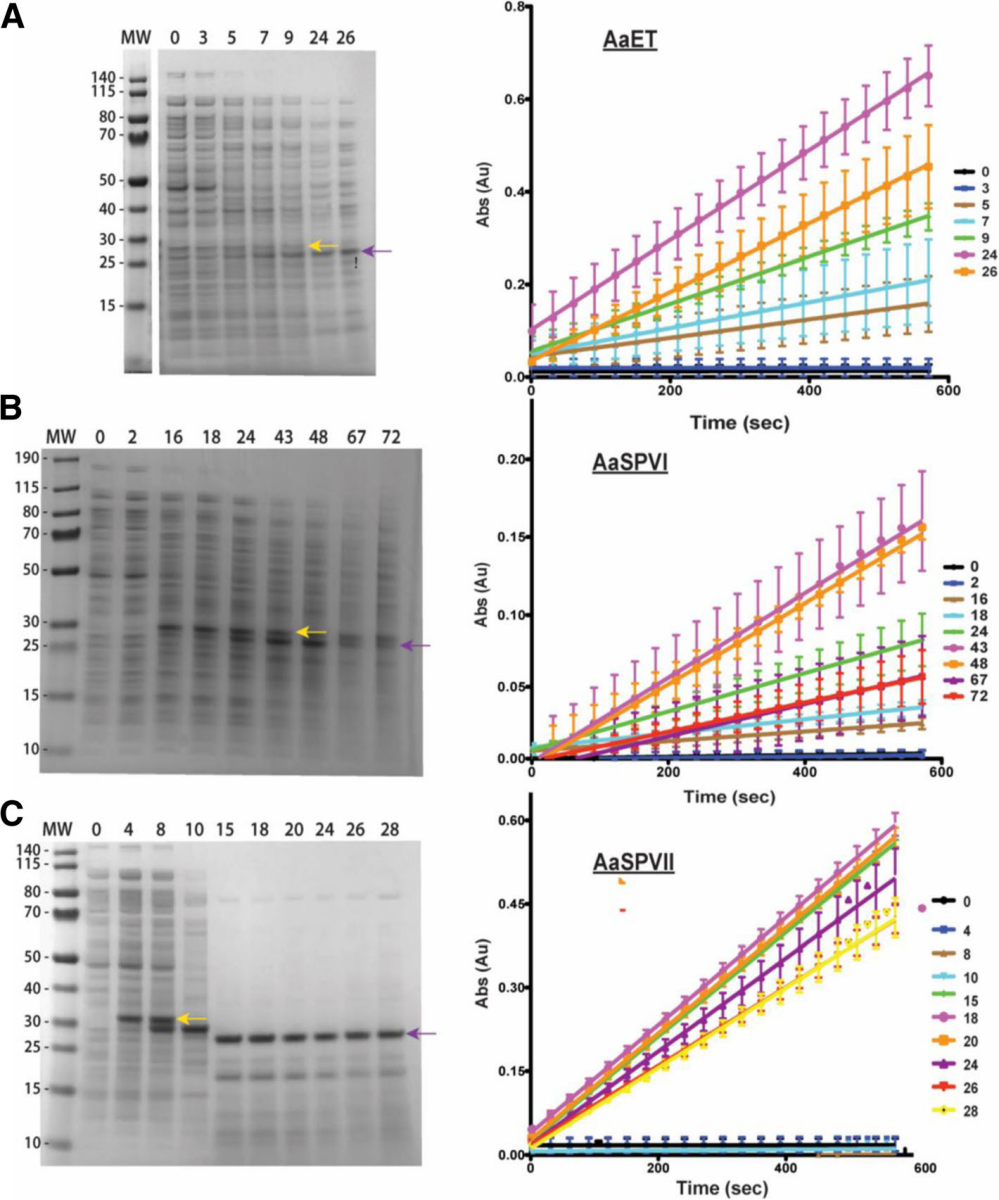
SDS-PAGE analysis and BApNA activity assays of samples collected from small-scale growth experiments of SHuffle® *E. coli* cells (NEB) grown in TB media at 15 °C (induced with 0.1 mM IPTG). Samples were collected at the given time points (in hours). The MW ladder is in kilo-Daltons (kDa). **a** The gel represents the soluble expression of AaET-NL zymogen (MW ~ 27.0 kDa, yellow arrow), auto-activating to the active mature form (MW ~ 22.4 kDa, purple arrow). The presence of active AaET at 5 h post-induction correlates with an increase in BApNA activity, with maximal activity observed at the 24 h time-point (plot on the right). **b** The gel represents the soluble expression of AaSPVI-NL zymogen (MW ~ 28.7 kDa, yellow arrow), auto-activating to the active mature form (MW ~ 24.1 kDa, purple arrow). The presence of active AaSPVI at 16 h post-induction correlates with an increase in BApNA activity, with maximal activity observed at the 67 h time-point (plot on the right). **c** The gel represents the soluble expression of AaSPVII-NL zymogen (MW ~ 28.7 kDa, yellow arrow), auto-activating to the active mature form (MW ~ 24.2 kDa, purple arrow). The presence of active AaSPVII at 15 h post-induction correlates with an increase in BApNA activity, with maximal activity observed at the 18 h time-point (plot on the right). Unlike AaET and AaSPVI, AaSPVII expression results in a species that lies between the zymogen and active forms starting at 8 h post-induction and disappearing at 15 h. This species is an inactive form of AaSPVII since no detectable BApNA activity observed

As for AaSPVI-NL and AaLT-NL, expression at 23 °C resulted in insoluble expression similar to the results in Fig. 2. Therefore, we attempted to grow the cells and express the proteases (including AaET-NL and AaSPVII-NL) at 15 °C (post-induction). The lower temperature slows down bacterial metabolism and the transcriptional/translational machinery, so longer incubation times are required to express the proteins of interest [21, 25, 27, 28]. For AaET-NL, samples were collected up to 26 h (post-induction), for AaSPVI-NL to 72 h, and for AaSPVII-NL 28 h. Surprisingly, all three proteases were expressed solubly but with the appearance of a second band over time, similar to the 23 °C expression of AaET-NL (Fig. 3a). We hypothesized that the second band might be the active form of each protease, and to test this, we utilized BApNA. Purified AaET, AaSPVI, and AaSPVII have been shown to proteolytically cleave BApNA releasing the p-nitroanilide chromophore [15]. Soluble crude lysates (20 μl) at pre- and post-induction samples were tested for BApNA activity, and simultaneously analyzed by SDS-PAGE. As seen in Fig. 4a, the zymogen form of AaET-NL is solubly expressed (as indicated by the yellow arrow) and begins to disappear at 5 h (post-induction) and a new more pronounced band begins to appear (purple arrow), which correlates with increasing BApNA activity. BApNA activity of AaET crude lysates is not observed until the 5 h time-point, reaching maximal activity at 24 h post-induction. For AaSPVI-NL, soluble expression is not observed until 16 h post-induction, with strong visualization of the active form (purple arrow) beginning at 24 h post-induction (Fig. 4b). BApNA activity is also observed, but delayed compared to AaET, starting at 16 h, followed by maximal activity at 67 h, and loss of activity at 72 h post-induction. Unlike AaSPVI-NL, soluble expression for AaSPVII-NL is observed at 4 h, with the active species appearing at 15 h post-induction, which correlates with detectable BApNA activity (Fig. 4c). Interestingly, an intermediate species (between the zymogen (yellow arrow) and the active form (purple arrow)) appears at 8 h, becomes the most prominent band at 10 h, and disappears at 15 h (gel in Fig. 4c). There is no detectable BApNA activity observed at these time-points, indicating an inactive zymogen species. There is a possibility that this protease may be cleaving at the Arg position in the thrombin cleavage site (LVPRGS) (see Fig. 1), before activating to the mature form. Work is currently underway to determine this intermediate species.

**Fig. 5:**
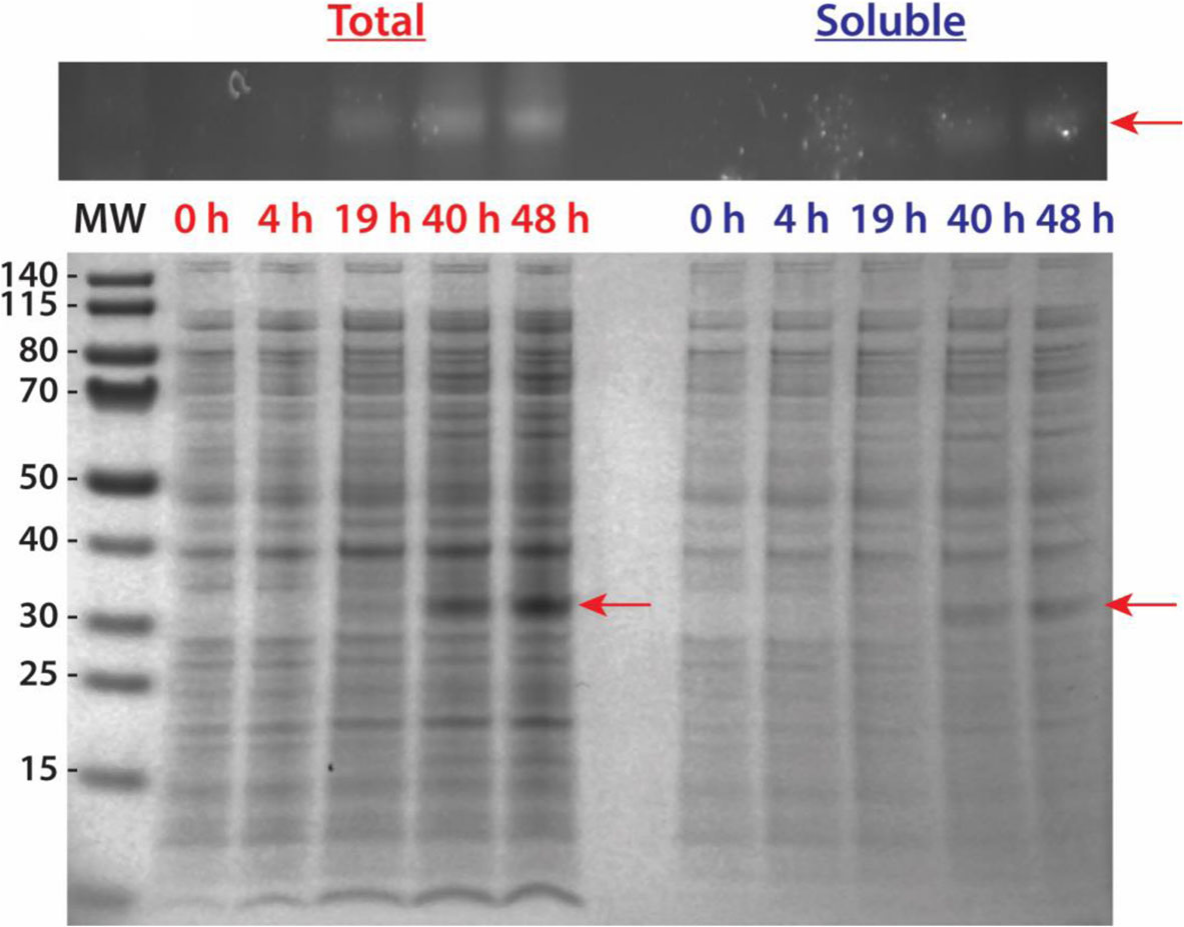
Large-scale soluble expression of recombinant AaLT-NL zymogen protease grown in TB media at 10 °C (induced with 0.1 mM IPTG). Plasmid construct was transformed into SHuffle® T7 Express Competent *E. coli* cells (NEB). Samples were collected at the given time points (in hours). The MW ladder is in kilo-Daltons (kDa). Gel analysis of samples collected from the growth of AaLT was first visualized using InVision™ His-Tag In-Gel Stain (Invitrogen), which specifically chelates to and enhances the fluorescence of poly his-tagged proteins (top figure). The His-Tag stain is the positive identification that the bands expressed in the gel below are indeed the expression of soluble AaLT-zymogen (MW ~ 27.6 kDa, red arrows). The growth was extended beyond 24 h due to the 10 °C growth conditions, which helped in solubly expressing the protease, but also to increase bacterial cell density in order to obtain a large quantity of cell paste for purification

With the successful expression of AaET-NL, AaSPVI-NL, and AaSPVII-NL at 15 °C, we decided not to repeat the growths at 10 °C. However, given that AaLT-NL was not solubly expressed at the above temperatures, 10 °C growth experiments were set, successfully producing solubly expressed protease (Fig. 5). For these experiments, InVision™ His-Tag In-Gel Stain was utilized to visualize the presence of the his_6_-tagged AaLT-NL protease. The colder temperature results in lower overall cell density and protease expression, see [21, 25, 27, 28], therefore the stain was useful in visualizing and confirming the presence of AaLT-NL. As shown in Fig. 5, soluble expression was observed at 19 h post-induction with maximal soluble expression observed at 48 h. Under these conditions, only a single band was observed. BApNA activity assays were not used since the protease does not to cleave BApNA in vitro and its protease specificity is currently unknown [15].

### Purification of Solubly expressed AaSPVII and AaLT zymogen (native propeptide) proteases

**Fig. 6:**
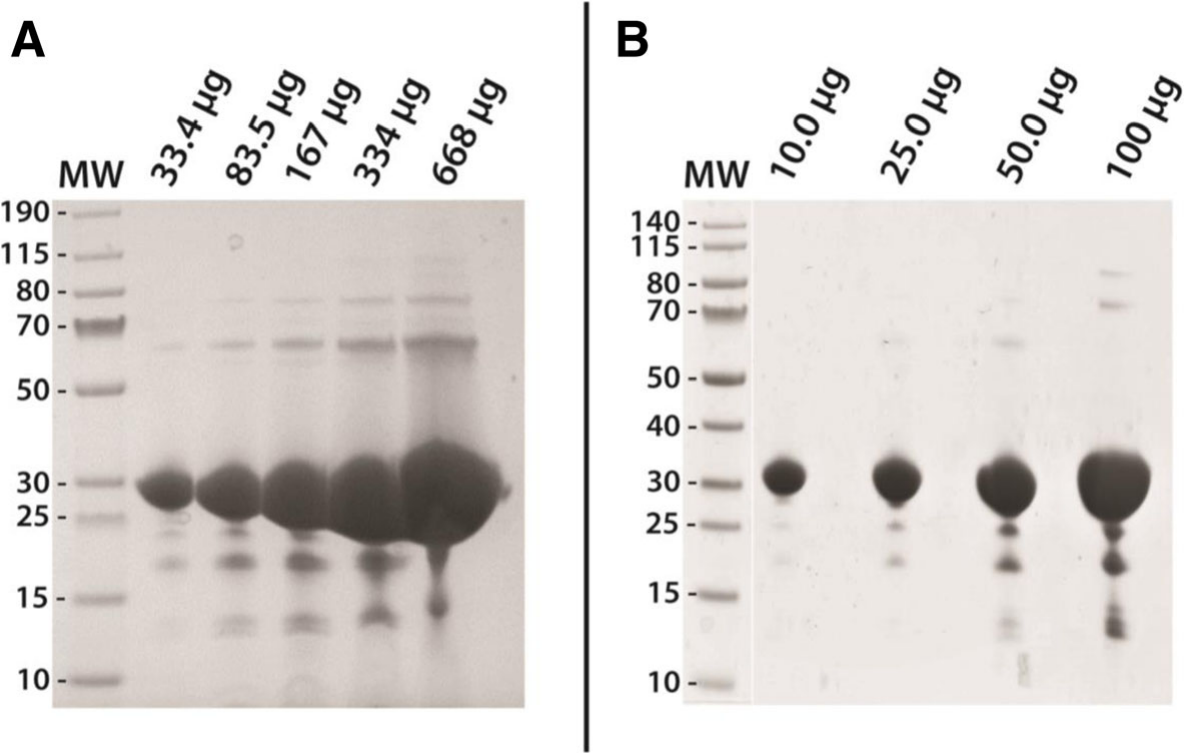
Final gel of Nickel purified recombinant zymogen (no leader) proteases grown and expressed at either 23 °C or 10 °C. In order to demonstrate that inactive zymogen proteases (with intact N-terminal his_6_-tag) can be isolated, AaSPVII grown at 23 °C (**a**) and AaLT grown at 10 °C (**b**) were purified. These samples are the post-dialysis concentrate of AaSPVI-NL and AaLT-NL dialyzed in 50 mM Sodium Acetate pH 5.2 + 2 mM DTT (buffer exchanged twice and set at 4 °C). Samples loaded on the gel are in micrograms (μg) and are increasing in order to show that the single step purification scheme led to a near homogenous sample with very little contaminants

With the successful soluble recombinant expression of the most abundant mosquito midgut proteases, and for the purpose of this study, we focused on the Nickel purification of AaSPVII and AaLT, two proteases expressed at different temperatures. The activation of mosquito midgut proteases is unknown and has been hypothesized that the zymogens might be auto-catalytic [29], see also Fig. 4. Therefore, to avoid potential activation of the proteases during clear lysate preparation and purification, the reducing agent dithiothreitol (DTT) was added to all buffers at a concentration between 1 to 2 mM. All buffers were kept on ice and purified using the FPLC, which facilitated fraction collection of the protease of interest. Immediately after identifying the protease fractions, all were pooled and dialyzed in Sodium Acetate buffer pH 5.2 in order to avoid unexpected potential auto-activation. Trypsins have been shown to auto-activate at pH 7.0 [30, 31], which is close to the purification buffer conditions used. This approach proved to be successful because, as seen in Fig. 6, a single band is observed in the single-step Nickel purification of both AaSPVII and AaLT. This is the final gel after purification, pooling of fractions, buffer exchange dialysis, and protease concentration. Very little contaminates are observed in each gel, but the proteases can be further purified and optimized using either ion exchange or hydrophobic interaction chromatography.

## Discussion

The necessary blood feeding behavior of the *Ae. aegypti* mosquito facilitates the transmission of potentially deadly and harmful viruses to uninfected human hosts. Zika, Dengue, Yellow Fever, and Chikungunya are mosquito-borne viral diseases that have become or are becoming a global health concern [4]. At the moment, there are no treatments, limited vaccines or therapeutics available to combat these mosquito-borne viral infections, which have led to high endemics and epidemics observed over the past few years [32]. Therefore, the only effective strategy still remains to be vector control, and with *Ae. aegypti* resistance to chemical compounds and effects to other insect species, new and more effective strategies are needed. A potential strategy may be to focus on blood meal digestion and the proteases involved in this process [1, 13–16]. With knockdown studies on three midgut proteases (AaSPVI, AaSPVII and AaLT) leading to a decrease in fecundity [1], may provide a potentially new vector control strategy. However, for this to be realized, in vitro biochemical studies focusing on midgut proteases must first be conducted. Even then, producing soluble recombinant mosquito proteases must first be achieved before these studies can be initiated.

Initial work to recombinantly express the zymogen (inactive) and mature (active) *Ae. aegypti* midgut proteases led to insoluble expression [15]. Furthermore, due to unknown activation of the midgut proteases, a strategy using a pseudo propeptide region with an unnatural enterokinase sequence was developed in order to facilitate activation of the proteases in vitro. To rescue the enzymes from inclusion bodies, a denaturation/renaturation scheme was developed [15]. Although this approach was successful in isolating bona fide active midgut proteases for initial enzyme kinetic analysis, the process is tedious and time consuming, and may not lead to yields comparable to proteins that can be solubly expressed [33]. In addition, the mode of activation of each zymogen is still unknown because the native propeptide region was removed [15]. Therefore, the purpose of this work is to describe the approach taken to recombinantly and solubly express the zymogen AaET, AaSPVI, AaSPVII, and AaLT midgut proteases with the native propeptide region using *E. coli* as the host. This will provide a much faster and facile starting point to researchers who have difficulty in producing solubly recombinant mosquito proteases in *E. coli*.

The field of Biochemistry has been revolutionized by the success of producing recombinant proteins using bacteria [20]. Without the molecular techniques, vast commercially available expression vectors, engineered bacterial strains, and rich media cultivation methods, large amounts of blood fed *Ae. aegypti* mosquitoes would be required to isolate midgut proteases that are only present once a mosquito has imbibed a blood meal [1, 15]. Because of the ease of manipulation, growth, and for institutions with limited funding, the cost effectiveness of recombinantly expressing proteins using *E. coli*, has made this organism the most widely preferred [24, 34]. Of course, there are limitations to recombinantly expressing proteins in *E. coli* (such as low expression, protein aggregation, plasmid instability, and protein degradation), but for each case there are available published troubleshooting strategies that address each potential problem (see [20, 24, 34]). As such, every protein to be recombinantly expressed will have its own problems and must be individually optimized to ensure the production of soluble and active protein. For the production of the most abundant zymogen midgut proteases, we have taken the troubleshooting ideas described in several review articles and highlight the most important parameters required to successfully producing soluble proteases using *E. coli*, which as we found were the type of bacterial cells and bacterial growth and induction temperature.

A major issue when attempting to recombinantly express eukaryotic proteins in bacteria is aggregation, especially proteases dependent on disulfide bridge formation for structure, stability, and function. Amino acid sequence analysis on AaET, AaSPVI, AaSPVII, and AaLT revealed the presence of six cysteine residues, which are predicted to form three disulfide bridges in each protease. It was not surprising that expression of these proteases in BL21(DE3) and Rosetta(DE3) bacterial strains led to insoluble protein (inclusion bodies) [15]. The cytoplasm of these *E. coli* strains are highly reducing and the reducing environment is caused by the thioredoxin and the glutathione/glutaredoxin reductase pathways, reducing disulfide bonds in proteases leading to misfolding [20, 33]. Furthermore, the initial attempt at expressing the zymogen midgut proteases included the protein leader (signal) sequence [15], a portion of amino acids on the N-terminus of the protein that is recognized and targeted to the endoplasmic reticulum for secretion into the cytoplasm. The problem, however, is that this polypeptide is usually hydrophobic in nature and has been shown to cause protein aggregation, thermodynamically destabilizing the recombinant expression of the protein of interest in *E. coli* [26]. To circumvent this issue, the signal sequence of each midgut protease was removed using PCR, keeping only the natural propeptide region (Fig. 1). This was a similar approach taken in the 2011 study where the signal sequence was removed when the unnatural EK site was introduced into the recombinant midgut proteases [15].

In order to avoid solubility expression issues resulting from improper disulfide bond formation, we turned to SHuffle® T7 Competent *E. coli* cells (NEB) [21]. These cells are BL21(DE3) derivatives that carry mutations in the reductase pathways (thioredoxin and glutathione/glutaredoxin) leading to a more oxidizing cytoplasm, which should allow formation of disulfide bridges [21]. In addition, the cells are engineered to constitutively express a disulfide bond isomerase (DsbC), a protein that aids in correcting of mis-oxidized disulfide bonds. Although the cytoplasmic conditions are ideal for promoting disulfide bridge formation, expression of the midgut proteases still led to insoluble expression when grown at the recommended 30 °C temperature. This was observed for all protease as seen in Fig. 2. This 30 °C temperature is 7 °C lower compared to wild type *E. coli*, BL21(DE3), and other closely related strains. Regardless, whether using BL21(DE3) or its derivatives, including SHuffle® T7 cells, when attempting to express eukaryotic proteins in a prokaryotic system there is a chance that at the optimal temperature, the eukaryotic proteins may be expressed insolubly. Because transcription and translation happen simultaneously in the bacterial cytoplasm, the rate of protein synthesis is approximately ten times faster than that of a eukaryotic cell [33]. And since a eukaryotic protein is being synthesized in a foreign prokaryotic environment, the rate of folding of the recombinantly expressed protease is not ideal. Prokaryotic proteins tend to fold at a much faster rate than their eukaryotic counterparts at the optimal growth conditions, and with the combination of a speedy rate of synthesis and slow folding in recombinant bacterial expression, the eukaryotic protein could aggregate and be insolubly expressed [35]. This provides a plausible explanation for why the midgut proteases were expressed insolubly at 30 °C in cells with a more oxidizing cytoplasm. To overcome this problem, we tested lower temperatures starting at 23 °C and going as low as 10 °C. Successful soluble expression was observed for AaET-NL and AaSPVII-NL at 23 °C (Fig. 3), but no soluble expression was observed for AaSPVI-NL and AaLT-NL. Interestingly, a second band was observed for AaET, which we hypothesized to be the active mature form. We attempted BApNA activity assays of the crude lysates, but since expression was only successfully visualized using WB, not enough solubly expressed AaET was present and could not reach the lower level of detection of p-nitroanilide formation (> 0.0125 abs units) [15].

The lower temperatures attempted (23 °C, 15 °C, and 10 °C) resulted in the soluble expression of all proteases (Figs. 3, 4 and 5). However, the preferred temperature differed for each. For example, AaLT-NL was only solubly expressed at 10 °C, while all the other proteases were solubly expressed at 15 °C. Importantly, at this temperature we were able to observe the possible auto-activation of AaET-NL, AaSPVI-NL, and AaSPVII-NL (Fig. 4). In each case, the presence of the active species (based on BApNA activity assays) seems to be dependent on protease concentration, which has been true for bovine and porcine trypsinogen [36]. In general, after induction with IPTG, protein expression concentration increases linearly with time at early time-points, but may reach a point where the expression is constant. This is the case for the midgut proteases. Expression of the zymogen form is initially observed, but over time, as expression concentration increases, leads to activation of the protease as observed in Fig. 4. At the moment, it is unknown if any proteases or enzymes in the bacterial crude lysates may be activating the recombinant midgut proteases, but the no induction growth experiment samples have no detectable BApNA activity (Additional files 1, 2 and 3: Figures S1, S2 and S3). Work is currently underway to determine if the midgut proteases are autocatalytic or if enzymes in the bacterial lysate are aiding in the process. Nonetheless, the reduced and colder temperature at which the proteases were expressed helped with promoting proper folding. By dropping the temperature at induction, the rate of protein synthesis, as well as the temperature-dependent hydrophobic interactions involved in protein folding are reduced, increasing the chances of proper folding when utilizing *E. coli* [37]. This temperature reduction approach led to successfully solubly expressing the four-zymogen (no leader) midgut proteases. It is important to note that caution should be taken when dropping the temperature lower than necessary because traditional promoter systems, bacterial transcription and translational machinery, and chaperones may not be as efficient compared to the optimal *E. coli* growth temperatures (37 °C or in the case of the SHuffle® cells, 30 °C) [20, 37]. Nonetheless, lowering the temperature at which recombinant proteases are expressed should be strongly considered before manipulation of any other variable.

Due to the length of time needed to solubly express the proteases of interest, we utilized Terrific Broth for all of our experiments. TB media contains yeast extract and tryptone at higher concentrations compared to LB, glycerol (an extra carbon source), and phosphate salts to help with culture acidification, making this much superior than LB [20]. In addition, the cell densities of the bacterial growths in TB are typically much higher compared to LB, which is important because the lower temperatures lead to a reduction in bacterial metabolism [25, 27, 28]. For each growth, the bacterial cells were grown at 30 °C to reach the proper induction at OD_600nm_ ~ 0.5–1.0 (this allows the density of the cells to increase at a much faster rate than if growing at the lower induction temperature), and then the temperature of the growths dropped to the determined value. At the colder temperatures, the length of time at which the cells are grown has to be extended, and as such the available nutrients may be depleted, not producing enough soluble protease. In general, *E. coli* growth in LB media stops at relatively low cell densities because of the limited nutrients and carbon sources [34]. Therefore, a richer media is preferred when growing at lower temperatures and for a longer extended period of time, which helped improve the amount of expression for all midgut proteases.

As a proof of principle, we proceeded to purify two zymogen proteases one expressed at 23 °C (AaSPVII-NL) and the other at 10 °C (AaLT-NL). With the observed possible auto-activation of the proteases, we wanted to ensure that halting and harvesting bacteria at an earlier time-point before the presence of the active species, would lead to successful purification and isolation of the inactive zymogen. This would be especially problematic for AaET, AaSPVI, and AaSPVII since auto-activation is observed (Fig. 4), but not problematic for AaLT since no auto-activation observed. We therefore, Nickel purified AaSPVII-NL (cells harvested at 5 h post-induction) and AaLT-NL (cells harvested at 48 h post-induction) to near homogeneity utilizing a modified three-step gradient approach to ensure separation of non-specific binding of proteins and our proteases of interest. The his_6_-tag was utilized in order to easily purify the proteases in one step, which as seen in Fig. 6, was achieved. Since the purification was done in the presence of DTT and cold (4 °C) buffer conditions, no auto-activation of the proteases was observed, even though the pH of the buffer was 7.2. Normally, this would be problematic since eukaryotic trypsins have been shown to auto-activate between pH 7 and 9 [30, 31]. Furthermore, to avoid any further auto-activation of the purified AaSPVII-NL and AaLT-NL zymogen proteases, buffer exchange dialysis into Sodium Acetate buffer pH 5.2 and protein concentration under these conditions did not lead to precipitation or loss of purified protease. More importantly, these conditions prevented auto-activation of the AaSPVII-NL zymogen protease. Work is currently underway to purify the other midgut zymogen proteases. Once we have isolated and purified all proteases, we will be able to determine the mode of activation and compare the kinetic parameters between solubly expressed recombinant proteases and the isolated refolded proteases from [15].

## Conclusions

The *Ae. aegypti* mosquito is an efficient biological vector capable of infecting more than one uninfected human host. The mosquito-borne viruses (Zika, Dengue, Yellow Fever, and Chikungunya) are easily transmitted through the blood feeding behavior of the mosquito, which is needed for the *Ae. aegypti* life cycle to continue. Midgut-specific proteases help digest blood meal proteins to produce the nutrients required for the gonotrophic cycle. With knockdown studies on three of the most abundant proteases (leading to effects on fecundity), inhibition of these and other midgut proteases may provide a new vector control strategy. However, before validating these proteases as inhibitor targets, further biochemical studies on the activation, activity, and specificity of each protease is needed. To achieve such goals, recombinant proteases must first be produced. The easiest and fastest system to produce recombinant protein is *E. coli*. However, the natural cytoplasmic conditions of most bacterial strains are reducing, which lead to improper folding of proteases dependent on disulfide bridges. Therefore, using a specialized strain of *E. coli* cells (SHuffle® T7 Competent cells, NEB) with a more oxidizing cytoplasm, we have been able to produce wild type (native) zymogen midgut proteases without the protein leader sequence. Furthermore, since bacterial expression has led to possible auto-activation, we have shown that halting and harvesting cells before the presence of the active species, can lead to the isolation and purification of the zymogens. The approach described here should provide researchers with a faster starting point to determine the ideal conditions for recombinant protease expression using *E. coli* as the host.

## Additional files

Additional file 1: **Figure S1.** SDS-PAGE analysis and BApNA activity assays of samples collected from the small-scale growth experiment of AaET-NL non-induced SHuffle® *E. coli* cells (NEB) grown in TB media at 15 °C. Samples were collected at the given time points (in hours). The MW ladder is in kilo-Daltons (kDa). The gel shows both the total and soluble samples collected at the same time-points as in Fig. 4a There is no expression of AaET-NL zymogen. In addition, very little to no BApNA activity is observed (plot on the right), similar to the pre-induction (0 h) and early post-induction (3 h) sample in Fig. 4a. (DOCX 556 kb)

Additional file 2: **Figure S2.** SDS-PAGE analysis and BApNA activity assays of samples collected from the small-scale growth experiment of AaSPVI-NL non-induced SHuffle® *E. coli* cells (NEB) grown in TB media at 15 °C. Samples were collected at the given time points (in hours). The MW ladder is in kilo-Daltons (kDa). The gel shows both the total and soluble samples collected at the same time-points as in Fig. 4b. There is no expression of AaSPVI-NL zymogen. In addition, very little to no BApNA activity is observed (plot on the right), similar to the pre-induction (0 h) and early post-induction (2 h) sample in Fig. 4b. (DOCX 1120 kb)

Additional file 3: **Figure S3.** SDS-PAGE analysis and BApNA activity assays of samples collected from the small-scale growth experiment of AaSPVII-NL non-induced SHuffle® *E. coli* cells (NEB) grown in TB media at 15 °C. Samples were collected at the given time points (in hours). The MW ladder is in kilo-Daltons (kDa). The gel shows both the total and soluble samples collected at the same time-points as in Fig. 4c There is no expression of AaSPVII-NL zymogen. In addition, very little to no BApNA activity is observed (plot on the right), similar to the pre-induction (0 h) and early post-induction (4, 8, 10 h) samples in Fig. 4c. (DOCX 746 kb)

## Abbreviations

AaET: *Aedes aegypti* Early Trypsin
AaLT: *Aedes aegypti* Late Trypsin
AaSPVI: *Aedes aegypti* Serine Protease VI
AaSPVII: *Aedes aegypti* Serine Protease VII
BApNA: Nα-benzoyl-D, L-arginine p-nitroanilide
