# Supplementary figures and images for: Soluble expression of recombinant midgut zymogen (native propeptide) proteases from the Aedes aegypti Mosquito Utilizing E. coli as a host
Source: BMC Biochem. 2018 Dec 18;19:12. doi: 10.1186/s12858-018-0101-0 (PMC6299515; doi:10.1186/s12858-018-0101-0)

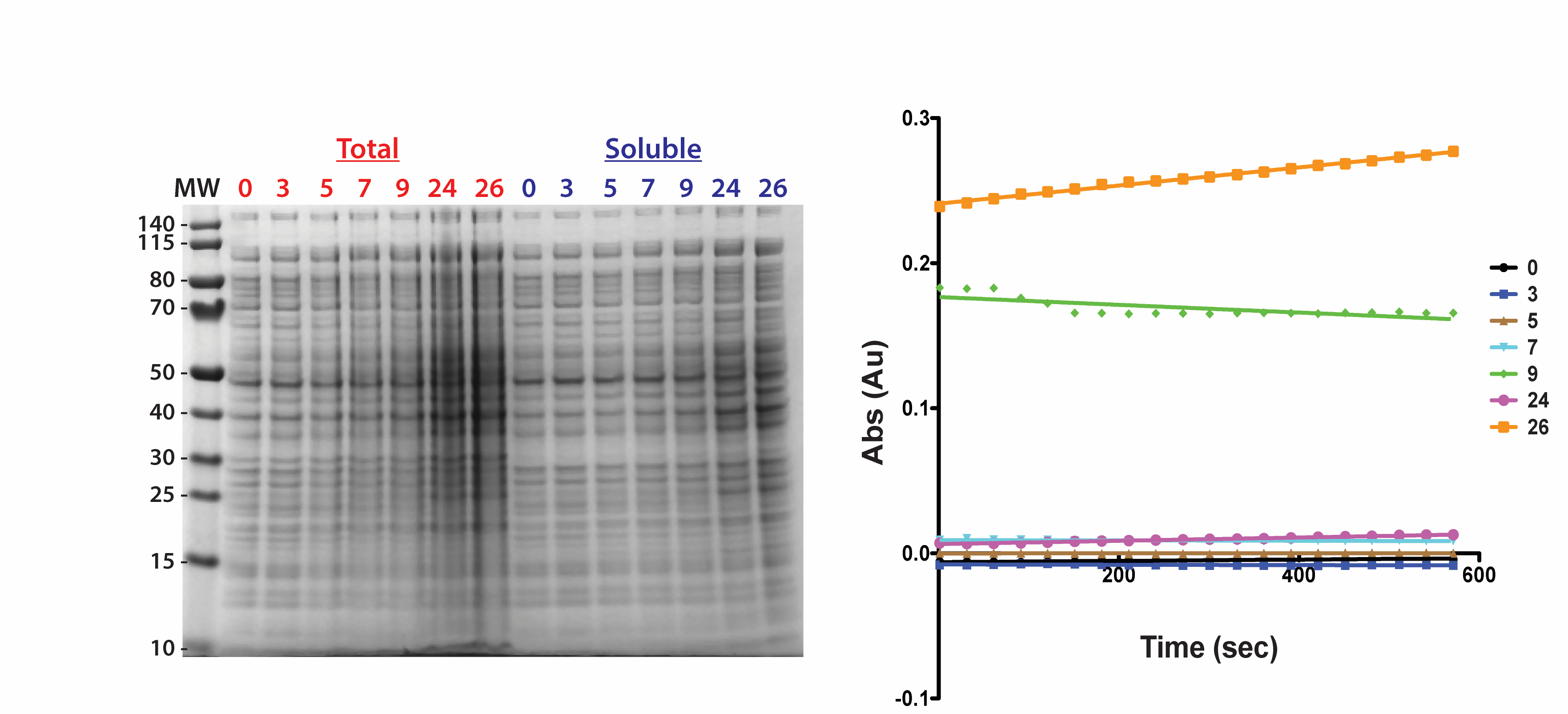

Supplement: Supplementary file 1 — Figure S1. SDS-PAGE analysis and BApNA activity assays of samples collected from the small-scale growth experiment of AaET-NL non-induced SHuffle® E. coli cells (NEB) grown in TB media at 15 °C. Samples were collected at the given time points (in hours). The MW ladder is in kilo-Daltons (kDa). The gel shows both the total and soluble samples collected at the same time-points as in Fig. 4a There is no expression of AaET-NL zymogen. In addition, very little to no BApNA activity is observed (plot on the right), similar to the pre-induction (0 h) and early post-induction (3 h) sample in Fig. 4a. (DOCX 556 kb) [file 12858_2018_101_MOESM1_ESM.docx]

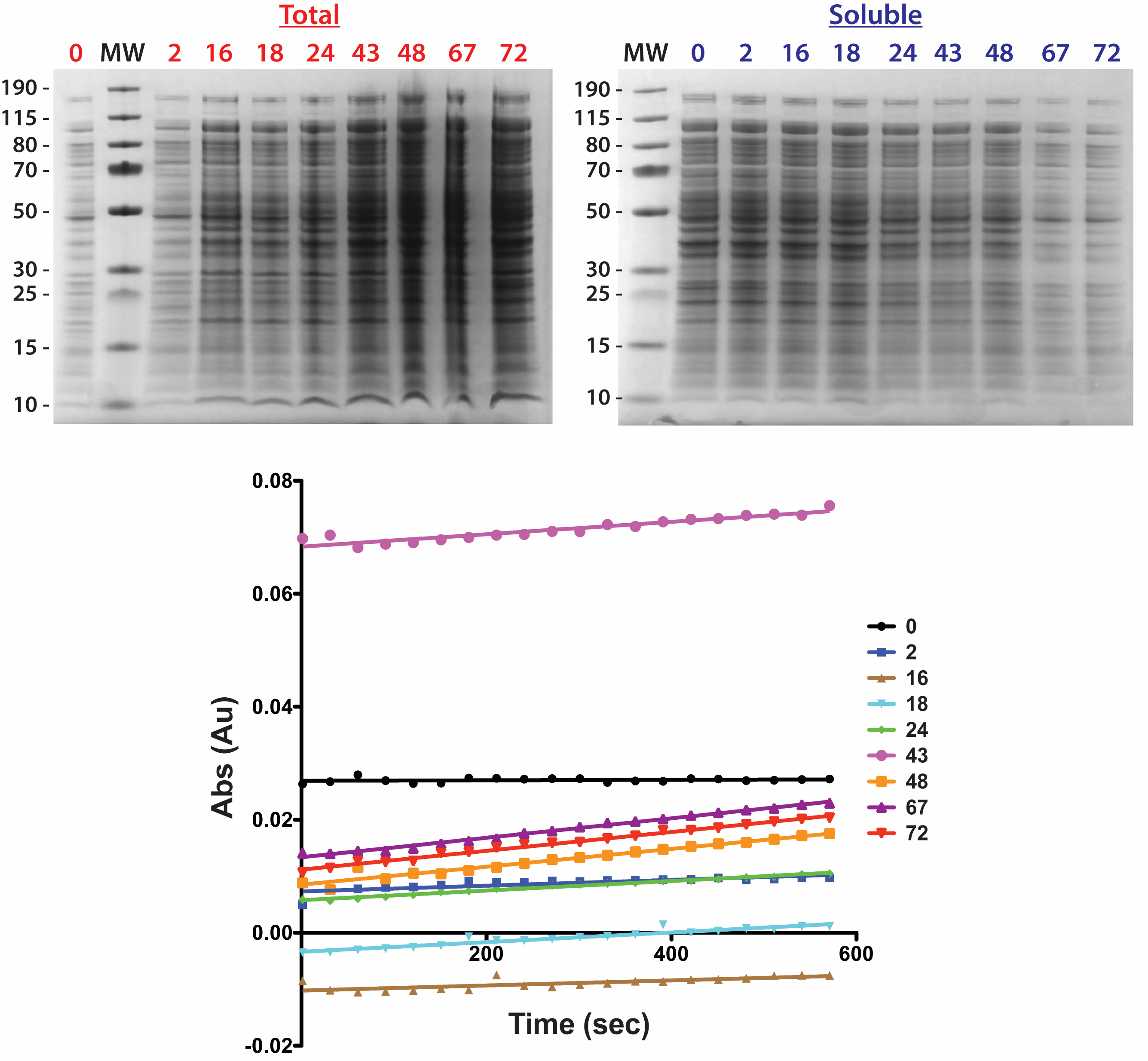

Supplement: Supplementary file 2 — Figure S2. SDS-PAGE analysis and BApNA activity assays of samples collected from the small-scale growth experiment of AaSPVI-NL non-induced SHuffle® E. coli cells (NEB) grown in TB media at 15 °C. Samples were collected at the given time points (in hours). The MW ladder is in kilo-Daltons (kDa). The gel shows both the total and soluble samples collected at the same time-points as in Fig. 4b. There is no expression of AaSPVI-NL zymogen. In addition, very little to no BApNA activity is observed (plot on the right), similar to the pre-induction (0 h) and early post-induction (2 h) sample in Fig. 4b. (DOCX 1120 kb) [file 12858_2018_101_MOESM2_ESM.docx]

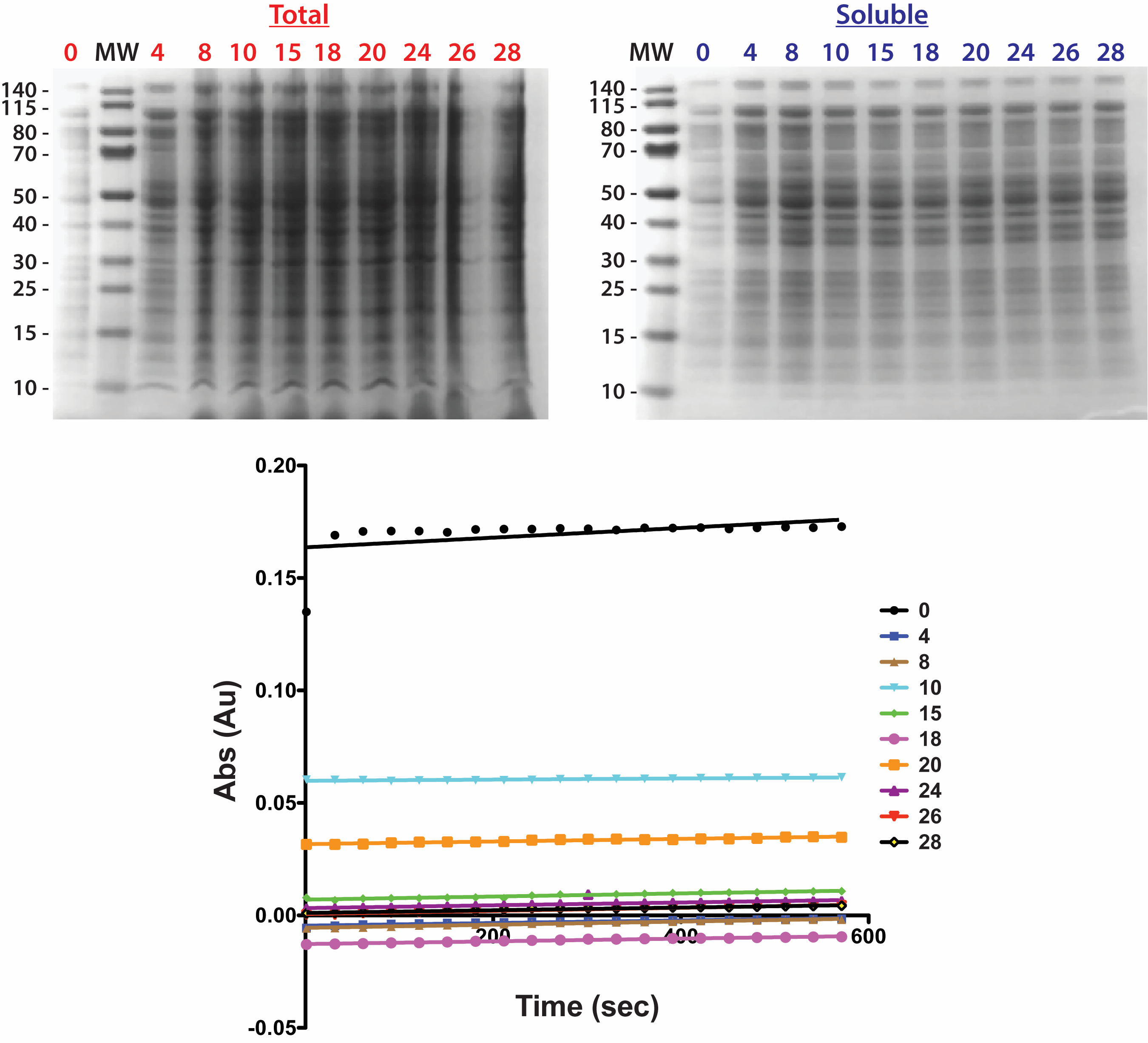

Supplement: Supplementary file 3 — Figure S3. SDS-PAGE analysis and BApNA activity assays of samples collected from the small-scale growth experiment of AaSPVII-NL non-induced SHuffle® E. coli cells (NEB) grown in TB media at 15 °C. Samples were collected at the given time points (in hours). The MW ladder is in kilo-Daltons (kDa). The gel shows both the total and soluble samples collected at the same time-points as in Fig. 4c There is no expression of AaSPVII-NL zymogen. In addition, very little to no BApNA activity is observed (plot on the right), similar to the pre-induction (0 h) and early post-induction (4, 8, 10 h) samples in Fig. 4c. (DOCX 746 kb) [file 12858_2018_101_MOESM3_ESM.docx]
